# The Role of T Cells in Ovarian Physiology and Infertility

**DOI:** 10.3389/fcell.2022.713650

**Published:** 2022-04-26

**Authors:** Laura O. Knapik, Shubangi Paresh, Dalileh Nabi, Lynae M. Brayboy

**Affiliations:** ^1^ University of New England, Biddeford, ME, United States; ^2^ Crimson Global Academy, Queen Creek, AZ, United States; ^3^ Department of Neuropediatrics Charité-Universitätsmedizin Berlin, Freie Universität Berlin, Humboldt-Universität zu Berlin, and Berlin Institute of Health, Berlin, Germany; ^4^ Department of Reproductive Biology, Bedford Research Foundation, Bedford, MA, United States

**Keywords:** T cells, ovary, female infertility, autoimmune disease, aging

## Abstract

Infertility affects one in six couples worldwide, with more than 48 million couples affected internationally. The prevalence of infertility is increasing which is thought to be attributed to delayed child-bearing due to socioeconomic factors. Since women are more prone to autoimmune diseases, we sought to describe the correlation between ovarian-mediated infertility and autoimmunity, and more specifically, the role of T cells in infertility. T cells prevent autoimmune diseases and allow maternal immune tolerance of the semi-allogeneic fetus during pregnancy. However, the role of T cells in ovarian physiology has yet to be fully understood.

## Introduction

### Age-Related Infertility

Infertility is a disease that impacts one in six couples worldwide. Approximately 48 million couples and 186 million individuals are affected by infertility globally (WHO, 2021). Although prevalent, little is known about the underlying contribution of age-related infertility. This topic is becoming increasingly important as many people opt to delay childbearing due to cultural and socioeconomic factors (Balasch and Gratacós, 2012). While there are some clinical interventions to circumvent age-related infertility, assisted reproductive technologies (ART) are largely inaccessible to much of the global population due to need for specially trained physicians, excessive cost, and extensive laboratory equipment with trained embryologists (Fleetwood et al., 2010; SART, 2021). Age-related decline in oocyte quantity and quality is observed in ovarian physiology, which can lead to infertility as women approach the climacteric. Some women may become prematurely infertile due to diminished ovarian reserve (DOR), which describes a decline in oocyte quantity earlier than physiologically expected (Pastore et al., 2018). The definition of DOR is varied and a precise etiology has yet to be identified. Patients diagnosed with DOR are sometimes unable to utilize ART to overcome infertility, as the disease is known to have a reduced response to ovarian stimulation (Ata et al., 2019). However, young women (<35 years old) with DOR have a better chance of pregnancy compared to aged counterparts, as the quality of the oocyte is not compromised, even though the quantity is. Therefore, age plays a quintessential role in infertility (Pastore et al., 2018). Despite technological advancements, the basic mechanisms of ovarian aging are still not fully understood, and even less is known about the interplay between the immune system and ovarian aging.

### Chronic Inflammation in Infertility

There are multiple theories suggesting a mechanism of decreased oocyte quality in relation to age (Ge et al., 2015). One widely held theory, the limited pool theory, states that oocyte quality is affected by an altered hormonal environment, which is thought to be caused in part by DOR. In the limited pool theory there is a smaller pool of oocytes and an altered hormonal environment, which may alter cellular function. For example increased levels of FSH in DOR might rescue defective oocytes and allow them to resume meiosis. Otherwise, these gametes would undergo apoptosis. However, because of FSH’s effects on microtubules, a higher FSH may enhance aneuploidy in oocytes (Dursun et al., 2006; Xu et al., 2011). Taken together, increased FSH allows oogenesis to progress in DOR, but at the cost of chromosomally abnormal oocytes. An alternate theory clashes with the limited pool theory, and states that oocyte quality is affected as physical damage accumulates over time (Zhang et al., 2020).

A less studied theory is the inflammatory theory of aging, dubbed “inflammaging” which has emerging data linking aging with significant immune cell populations in the ovary (Franceschi and Campisi, 2014; Briley et al., 2016). Inflammaging is human aging characterized by low-grade, chronic inflammation (Franceschi and Campisi, 2014). It can be explained using the free radical theory of aging, which illustrates the negative impact of aging on the body (Fulop et al., 2014). Free radical theory of aging states that free radicals, from the environment as well as internal metabolism, cause oxidative damage to cellular elements which, over time, results in an accumulation of structural and functional errors in the body (Pomatto and Davies, 2018). The ovaries are not excluded from this process, and are susceptible to inflammatory damage.

There is emerging evidence regarding resident immune cell populations within the ovary (Briley et al., 2016; Zhang et al., 2020). Previous study has hypothesized early development of a limited pool of ovary-committed bone marrow cells during embryogenesis (Bukovsky and Caudle, 2012). Once depleted, a process that can occur physiologically through aging or pathologically (ie following induction of chemotherapies), oogenesis and follicular restoration are unable to proceed. This demonstrates a fundamental link between ovarian health and the immune system. In addition to aging other examples of chronic inflammation, such as obesity-related inflammation, affect ovarian function as well as oocyte quality (Snider and Wood, 2019). Autoimmune diseases, which by definition are highly inflammatory processes, have been linked to DOR (Sharif et al., 2019). An estimated 10–40% of women with ovarian insufficiency are also diagnosed with an autoimmune disease (Sharif et al., 2019). An astounding 40% of patients diagnosed with autoimmune polyglandular syndrome type 1 (APS-1) are also diagnosed with DOR. APS-1 is a multi-organ, autosomal recessive mutation of the autoimmune regulator gene (AIRE) that directly dysregulates T cell function. Given this link between immunity, specifically T cell failure, and DOR, it is critical to further elucidate the role of T cell biology in the ovary.

### T Cells in the Ovary

T cells are resident white blood cells present in the body and the ovary, but very little has been reported about their physiologic role in normal ovarian function. T cells are derived from the bone marrow and are further developed in the thymus to supply the periphery with mature, self-replicating T cells (Hong, 2001). Specifically, the thymus provides a location for antigen presenting cells (APCs) to present antigenic material to naïve T cells, thus promoting T cell maturation and the cell’s ability to distinguish between self and non-self antigens, a process known as tolerance (Krueger et al., 2017; Verma and Kelleher, 2017). There are three subsets of T cells: T helper cells, cytotoxic cells and regulatory T cells (Tregs), that all function as critical effectors of the immune response (Gagliani et al., 2017).

Immune cells, particularly Tregs, play a crucial role in the tolerance of allogeneic pregnancy- related tissues and autologous oocytes (Guerin et al., 2009). Maternal immune tolerance of paternal MHCs is important for pregnancy success and fetal survival, as there is evidence of activation against male gametes by the maternal system (Guerin et al., 2009). Therefore, Treg cells may prevent miscarriages that are caused by maternal rejection in humans (Saito et al., 2007). There is evidence demonstrating Tregs in the ovaries are more potent suppressors of autoimmunity than in Tregs in males (Hong, 2001). In a mouse model, when the thymus was removed shortly after birth, spontaneous ovarian autoimmune disease occurred (Samy et al., 2006). Dysregulation of Tregs is thought to be the basis of autoimmune disease establishment and advancement. Autoimmune diseases tend to be more prevalent in women compared to men, and approximately 80% of patients diagnosed with autoimmune diseases identify as female (Ngo et al., 2014; Angum et al., 2020). In thyroidectomized female mice, CD4^+^ and CD25^+^ T cell rescue effectively inhibited autoimmune ovarian disease progression compared to thyroidectomized controls (Alard et al., 2001). Clinically, autoantibodies in the ovary are commonly associated with unexplained infertility (Luborsky, 2002). The ovaries are not an immunologically privileged organ and so, a slight variation in tolerogenic, ovary-specific, antigens can have significant negative effects on fertility (Warren et al., 2014). Interestingly, there is a conspicuous population of T cells within the ovary. In a previous study, ovarian cortex samples from 21 healthy, human patients of reproductive age (20–37 years of age) were analyzed via single cell mRNA sequencing for markers selected to identify distinct cell populations (Wagner et al., 2020). Within the study, a small population of immunologic cells were identified, including markers specific to T cells. This finding demonstrates the presence of immunologic cells, such as T cells, within the physiologically competent ovary. An additional study using tissue samples from the medulla of five human ovaries identified a significant cluster of T cells, which constitute approximately 6.2% of the ovary, through single cell RNA sequencing (Wagner et al., 2020; UCSC, 2022). A recent elegant body of work by Ma et al. extrapolated the ovarian gene expression of young (5–6 months) and aged (21–22 months) mice to further identify markers of ovarian aging. Within this study, a significant proportion of differentially expressed genes between the two populations were related to immune function, and aged mice had significantly lower values of naïve CD4^+^ T cells compared to their younger counterparts. This provides further evidence that naïve CD4^+^ T cells play a fundamental role in ovarian aging, and decreased presence may represent decreased ability to mount an immune response, perhaps contributing to diminished fertility associated with age (Ma et al., 2020). Taken together, these studies indicate an exigent need to understand the mechanism of T cell regulation in the ovary, as there are potential implications for ovarian disease and fertility outcomes.

## T Cells in Ovarian Disease

There is a paucity of data regarding the normal, physiologic mechanism of T cell activity in the ovary. However, there is evidence that T cells are involved in the pathogenesis of several chronic ovarian diseases. It is important to note that it is difficult to distinguish the T cells presence as causal or reactive in both autoimmune processes or metabolic disorders that impact the ovary. Herein we describe some of these disorders:

### Autoimmune Oophoritis

Autoimmune oophoritis is a rare, autoimmune disorder resulting in destruction of the ovary that clinically presents with amenorrhea and infertility (Jacob and Koc, 2015). Approximately 5% of people diagnosed with DOR are suspected to carry an autoimmune oophoritis diagnosis (Silva et al., 2014). Diagnosis is made by detecting antibodies in the serum that act against ovarian tissues, in part because the ovaries are difficult to access without surgical intervention (Warren et al., 2014). Very little has been elucidated regarding the mechanism, and specific antigenic target of autoimmune oophoritis. In an animal model, transgenic expression of maternal antigen that embryos require (MATER) was unable to completely prevent autoimmune oophoritis disease progression, suggesting multiple antigenic targets are at play (Otsuka et al., 2011). Histological study has revealed T cells are present in the inflammatory infiltrate of inflicted ovaries (Silva et al., 2014). This demonstrates a need to further understand normal T cell biology, and how this process may go awry resulting in autoimmune oophoritis.

### Polycystic Ovary Syndrome

Despite polycystic ovary syndrome (PCOS) being the most common endocrine disorder in women, with a prevalence ranging from 6 to 15% depending on the diagnostic criteria employed, a clear etiology for this heterogeneous disease has yet to be determined (Fauser et al., 2012). The diagnosis of PCOS is a diagnosis of exclusion that must have two out of three consensus criteria including: hyperandrogenism, anovulation or oligovulation, or characteristic ovarian morphology on ultrasound (Legro et al., 2013). PCOS is associated with infertility, 70–80% of individuals diagnosed with the disease struggle with diminished fertility (Ata et al., 2019). However, many organ systems are also impacted, and the overall health of a person with PCOS is compromised throughout their reproductive lifespan. Therefore, PCOS may represent a model for how an ovarian manifestation of a disease may foreshadow somatic disease and long-term non-reproductive clinical sequelae. There have been some reports associated with T cells in the ovaries of patients that suffer from polycystic ovarian syndrome. Specifically, reports of abnormal T cell activation and cytokine production were detected in the follicular fluid of patients diagnosed with PCOS compared to non-PCOS healthy controls (Li et al., 2019).

### Ovarian Cancer

Ovarian cancer is the most lethal gynecologic malignancy and is defined as an immunogenic tumor that triggers a natural antitumor immune response against it (Wang et al., 2018). Numerous papers have published a relationship between the ovary and T cells in ovarian cancer, specifically implying that T cells are heavily implicated in ovarian dysgenesis. However, few, if any papers address how T cells could be involved in the molecular mechanism of carcinogenesis of the ovary. One suggested mechanism is that tumor-specific antigens, processed via antigen presenting cells, are presented to T-cells which then activate CD8^+^ cytotoxic T-cells and CD4^+^ helper T-cells, which then infiltrate in the tumor. CD8^+^ cytotoxic T-cells directly target tumor cells whereas, CD4^+^ helper T-cells regulate an immune response by secreting cytokines that stimulate other immune cells (Westergaard et al., 2019). Thus, tumor-infiltrating T-cells, particularly CD4^+^ and CD8^+^ T cells are important predictors of ovarian cancer.

## Discussion

Given the burgeoning data suggesting the role of T cells in the ovary, it seems increased discussion and collaboration are needed to understand the complexity of the ovarian immunologic microenvironment. While *in vitro* fertilization (IVF) has afforded relief for many individuals with involuntary childlessness, it must be said that IVF, by all intents and purposes, is a primitive remedy in that it is unable to remediate previously damaged oocytes, potentially including oocytes that may be altered after residing in an immunologically compromised ovary. Further, immunological maladies impacting the ovary lead not only to infertility, but long-term health sequelae that impact women. Therefore, further funding and study is needed to examine the role of T cells in the normal ovarian physiology so that we can manipulate these processes to improve not only reproductive outcomes, but also improve the health of people with ovaries.
